# Effect of pueraria on left ventricular remodelling in HFrEF: A systematic review and meta-analysis

**DOI:** 10.1371/journal.pone.0295344

**Published:** 2023-12-04

**Authors:** Lipeng Shi, Lumei Huang, Erqian Yin, Jingwei Deng, Xuqin Du

**Affiliations:** 1 College of Traditional Chinese Medicine, Chongqing Medical University, Chongqing, China; 2 Department of Cardiovascular UnitUnit, Traditional Chinese medicine hospital Dianjiang Chongqing, Chongqing, China; 3 Chongqing College of Traditional Chinese Medicine, Chongqing, China; 4 Chongqing Traditional Chinese Medicine Hospital, Chongqing, China; Tehran University of Medical Sciences, ISLAMIC REPUBLIC OF IRAN

## Abstract

**Background:**

Heart failure with reduced ejection fraction (HFrEF) is a prevalent cardiovascular disease globally, posing a significant burden on healthcare and society. Left ventricular remodelling is the primary pathology responsible for HFrEF development and progression, leading to increased morbidity and mortality. Pueraria, a traditional Chinese herbal medicine and food, is commonly used in China to treat HFrEF. Accumulating evidence suggests that pueraria can effectively reverse left ventricular remodelling in HFrEF patients. This meta-analysis aims to assess the impact of pueraria on left ventricular remodelling in HFrEF patients.

**Methods:**

Eight electronic databases, including PubMed, EMBASE, Clinicaltrials.gov, Cochrane Library, Wanfang, CNKI, CQVIP, and CBM were searched for literature from inception to June 2023. All randomized controlled trials (RCTs) using pueraria in the treatment of HFrEF were included. The Cochrane Risk of Bias tool was utilized for RCTs’ methodological evaluation, while Review Manager 5.4.1 was used to analyze the data.

**Results:**

Nineteen RCTs with a total of 1,911 patients (1,077 males and 834 females) were identified. Meta-analysis indicated that combination medication of pueraria and conventional medicine (CM) was superior to the CM alone in raising left ventricular ejection fraction (LVEF; MD = 6.46, 95% *CI*, 4.88 to 8.04, *P* < 0.00001), and decreasing left ventricular end-diastolic diameter (LVEDD; MD = -4.78, 95% *CI*, -6.55 to -3.01, *P* < 0.00001), left ventricular end-Systolic diameter (LVESD; MD = -3.98, 95% *CI*, -5.98 to -1.99, *P* < 0.00001) and N-terminal pro-brain natriuretic peptide (NT-proBNP; MD = -126.16, 95% *CI*, -185.30 to -67.03, *P* < 0.0001). Besides, combination medication improved clinical efficacy rate (RR = 3.42, 95% *CI*, 2.54 to 4.59, *P* < 0.00001), 6-min walk test (6-MWT; MD = 65.54, 95% *CI*, 41.77 to 89.31, *P* < 0.00001), and TCM syndrome score efficacy (RR = 3.03, 95% *CI*, 1.57 to 5.83, *P* = 0.0009). Regarding safety, no difference was observed for adverse events (RR = 0.59, 95% *CI*, 0.22 to 1.54, *P* = 0.28).

**Conclusion:**

The use of pueraria combined with conventional medicine in HFrEF patients has superiority over conventional medicine alone in ameliorating cardiac function and reversing left ventricular remodeling. Moreover, combination medication has no increase in adverse drug events. Given some limitations, more prudence and high-quality clinical trials are needed in the future to verify the conclusions.

## Introduction

Heart failure (HF) is a chronic clinical syndrome with high mortality. It is characterized by cardiac dysfunction that results in a decrease in cardiac output and an inability to meet the body’s metabolic needs [1]. Defined as a global pandemic, there are over 6.5 million people in the United States and an estimated global prevalence of 64.34 million individuals with HF [1–3]. Despite medical advances, the occurrence of heart failure, specifically heart failure with reduced ejection fraction (HFrEF), has gradually increased over the past 30 years. This has placed a significant burden on healthcare and social systems [3]. Left ventricular (LV) remodeling refers to changes in LV structure, which are characterized by changes in LV size, shape, and a reduction in LVEF [4]. LV remodeling is the primary pathology of HFrEF occurrence and development, and it is also a key factor that influences the morbidity and mortality of HFrEF [5]. Therefore, it prompts clinicians and researchers to explore effective therapies for ameliorating LV remodeling in HFrEF patients.

In China, Chinese herbal medicine (CHM) is widely used as an adjunctive therapy for the treatment of HFrEF, and it has been verified to enhance efficacy and reduce side effects [6, 7]. Of particular research interest is the herb *pueraria* (*Gegen* in Chinese), which has extensive pharmacological actions, including antioxidant stress, anti-inflammation, hepatoprotection, renoprotection, and good cardioprotective effects [8–10]. Pueraria, sourced from the dried root of *Pueraria lobata* (Willd.) Ohwi, has been recognized as a homologous medicine and food and is widely used to treat HFrEF in China. It is reported that pueraria and its main bioactive component puerarin can effectively ameliorate cardiac function and reverse LV remodeling [11, 12]. Currently, there are only a few clinical trials conducted solely on pueraria for HFrEF [13–16], but there are several trials on CHM compound including pueraria as the main components for HFrEF [17–31]. These trials indicate that pueraria has exhibited positive therapeutic effects on LV remodeling in HFrEF patients. To date, no systematic studies have been conducted on the impact of pueraria on LV remodeling in HFrEF patients. Based on the available trials, we performed this meta-analysis to thoroughly evaluate the clinical efficacy and adverse drug events of pueraria in treating LV remodeling in HFrEF patients.

## Methods

The preferred reporting items for systematic reviews and meta-analysis (PRISMA) were adopted for guiding this study [32]. The study protocol was registered on PROSPERO (registration number: CRD42022330748).

### Search strategy

Eight electronic databases were systematically searched for literature from inception to June 2023: PubMed, EMBASE, Clinicaltrials.gov, Cochrane Library, Wanfang Database, China National Knowledge Infrastructure (CNKI), Chinese Science and Technology Journals (CQVIP), and Chinese Biomedical Literature database (CBM). The retrieval languages were limited to English and Chinese. Our review did not place any restrictions on publication date, or publication status. The search keywords included “pueraria”, “puerarin”, “kudzu”, “pueraria lobata”, “heart failure”, “heart dysfunction”, “ventricular dysfunction”, “heart insufficiency”, “randomized controlled trial”, “clinical trial”, “random”, “double-blind”, “single-blind”, and “trial”. S1 Text describes detailed search strategies.

### Inclusion criteria

#### Study design

RCTs on pueraria for HFrEF treatment were included, regardless of the publication type.

#### Participants

Only patients who met the recognized diagnostic of HFrEF, while maintaining LVEF<40%, were included in the study [33–36]. Primary diseases included hypertension, coronary heart disease, rheumatic heart disease, dilated cardiomyopathy, etc. Studies were not restricted by testee’s gender, age, race, or source.

#### Interventions

Patients in the experimental group were given a combination treatment of pueraria and controls. Regardless of administration route, various forms of pueraria were considered, including capsules, decoctions, tablets and injection.

#### Control

According to HF guidelines [33–36], the control group was administed with conventional medicine for HFrEF, including diuretics, ACEI, ARB, beta-blockers, spironolactone, and myocardial metabolism-improving drugs, etc.

#### Outcomes

Primary outcomes including: 1) Left ventricular ejection fraction (LVEF), 2) Left ventricular end-diastolic diameter (LVEDD), 3) Left ventricular end-systolic diameter (LVESD). Secondary outcomes including: 1) Clinical efficacy rate: clinical symptoms and signs obviously improved, meanwhile New York Heart Association (NYHA) increased over one grade, 2) N-terminal pro-brain natriuretic peptide (NT-proBNP), 3) Six-Minute walk test (6-MWT), 4) The TCM syndrome score efficacy: according to the standards established by Chinese health ministry. Safety outcome including: Adverse drug events: any untoward medical events that occurred during treatment.

### Exclusion criteria

The following conditions were employed to exclude studies: 1) study design: reviews, case reports, cell or animal studies, and uncontrolled trials; 2) participants: patients with severe infection, severe heart failure, malignant arrhythmias, hypothyroidism and severe liver and kidney failure; 3) Republished studies (only the most comprehensive data should be selected); 4) Due to incorrect or incomplete data, the meta-analysis cannot be integrated; 5) The full text could not be accessed online or through correspondence with the corresponding author via email.

### Data extraction and quality assessment

Endnote 20.5 was used to manage and screen records from databases. Two reviewers independently evaluated the articles based on inclusion and exclusion criteria. The third reviewer resolved any disagreements. Data extraction was simplified by using a standard form, including: 1) study ID (title, first author’s name, and publication year), 2) sample size, 3) patient characteristics (age, gender, and NYHA, etc.), 4) intervention details (dose, treatment duration and frequency of administration, etc.), 5) outcomes, adverse reactions, and risk of bias information. Quality assessment was independently carried out by two reviewers using the Cochrane Handbook 5.1 [37]. The evaluation mainly including: 1) random sequence generation method; 2) distribution concealment of randomization scheme; 3) blinding; 4) integrity of outcomes; 5) reporting bias; 6) other bias. After that, the bias risk assessment chart drawn was assessed and graphed using Review Manager 5.4.1 software.

### Data analysis

Review Manager software (version 5.4.1) was adopted for data analysis. Dichotomous variables were presented as risk ratio (RR), and continuous variables were presented as mean difference (MD). For both variables, 95% confidence intervals (CIs) were calculated. Based on the value of inconsistency index (*I*^2^), heterogeneity was statistically assessed and they were displayed in forest plots. A random-effect model was adopted for substantial heterogeneity (*I*^2^ ≥ 50%, or *P*<0.05); Otherwise, a fixed-effect model was applied. Subgroup analysis of primary outcomes (LVEF, LVEDD, LVESD) was conducted depending on the combinations of pueraria and other herbs. The publication bias was analyzed, and it was reported in a funnel plot. In this study, statistical significance was determined by *P* value<0.05.

## Results

### Search results

**Fig 1:**
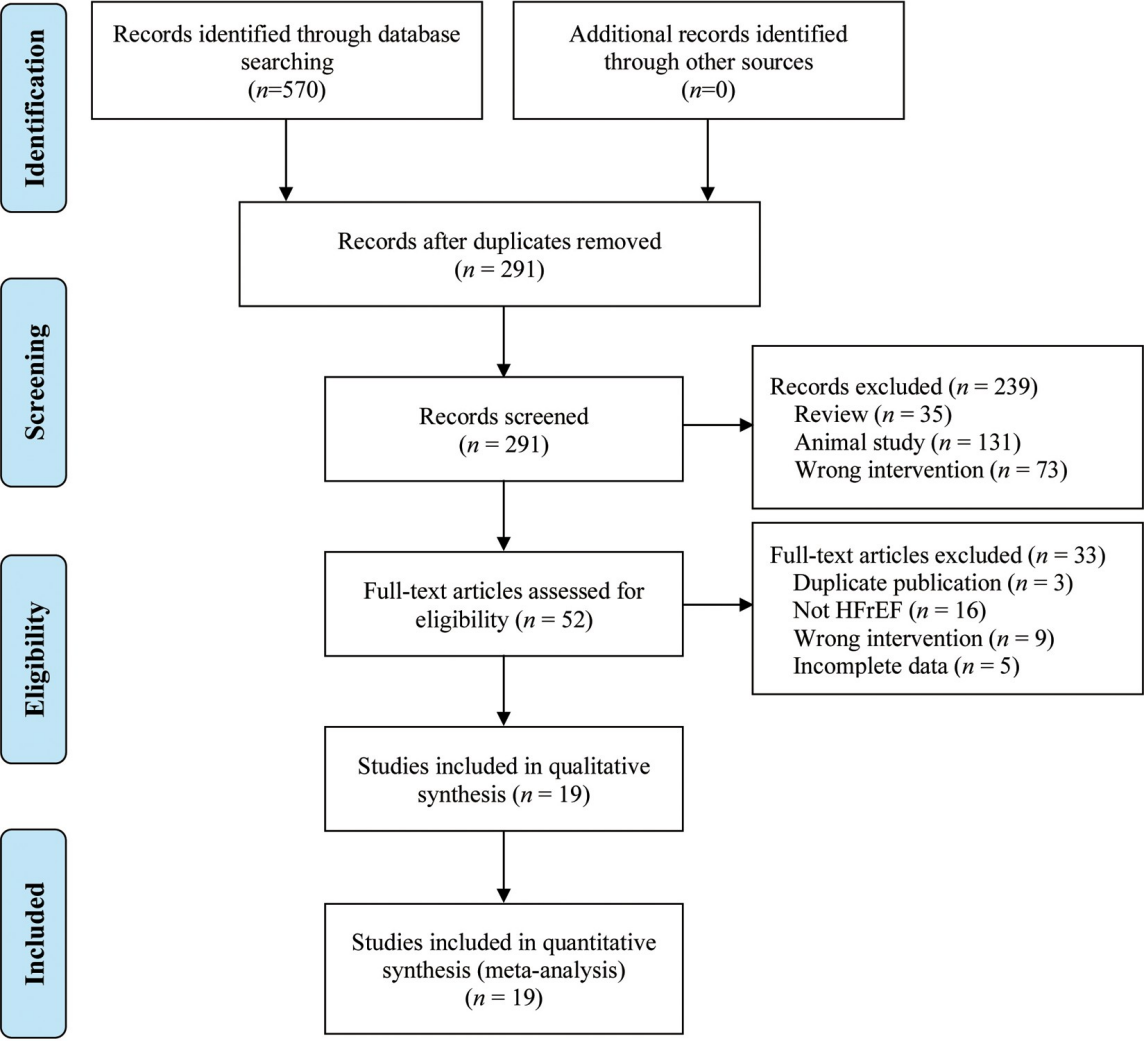
Study flow diagram.

Fig 1 showed our literature search’s result, which is conducted on June 2023. A total of 409 studies were identified by searching PubMed (*n* = 8), EMBASE (*n* = 9), Clinicaltrials.gov (*n* = 0), Cochrane Library (*n* = 1), Wanfang (*n* = 95), CNKI (*n* = 270), CQVIP (*n* = 102) and CBM (*n* = 85). As a result of reviewing the titles and abstracts, 239 studies were excluded. Following a full-text reading, 63 studies were excluded, and 19 studies [13–31] were finally included in quantitative synthesis.

### Study characteristics

**Table 1** presented 19 trials’ characteristics. A total of 1,911 patients (1,077 males and 834 females) participated in the study, and sample sizes ranged from 30 to 100. The control group was given conventional medicine for HFrEF, including diuretics, ACEI, ARB, beta-blockers, spironolactone, and myocardial metabolism-improving drugs, etc. The treatment group received pueraria combined with conventional medicine. Pueraria CHM compounds used in the included studies were Yangxinshi tablets, Getong Tongluo capsules, Dange Wuling powder, Tongyang Lishui decoction, Yiqi Yangyin Tongmai decoction, Xinshuning capsules, Linggui Zhugan decoction, and, Yixintong capsules. The intervention duration ranged from 10 days to 12 months. 18 studies [13–16, 18–31] reported LVEF, 9 studies [14, 15, 18, 19, 21, 24, 26, 28, 30] reported LVEDD, 7 studies [14, 15, 18, 19, 24, 26, 30] reported LVESD, 15 studies [14–19, 21, 23, 25–31]reported clinical efficacy rate, 4 studies [19, 25–27] reported NT-proBNP, 8 studies [18, 20, 22–27] reported 6-MWT, and 4 studies [20, 24, 26, 27] reported TCM syndrome score efficacy. Among the included studies, 10 studies [13–16, 19, 22, 24, 26–28] reported adverse events, 4 studies [13–15, 28] of them reported no adverse events occurred. Only 1 study [26] described the follow-up period.

**Table 1:** Basic characteristics of included studies.

Study ID	Sample size		Mean age (years)		Sex (M/F)		Diagnostic criteria	NYHA classification		Intervention measures		Treatment duration	Outcomes
	T	C	T	C	T	C		T	C	T	C		
An et al. (2016)	50	50	72.5	71.8	27/23	25/25	NR	II:10;III:19;IV:21	II:7;III:26;IV:17	CM + Xinkeshu tablets (1.24g, tid)	CM	4w	④
Cen et al. (2020)	45	45	54.9±10.2	53.9±10.2	24/21	23/22	NR	III:26;IV:19	III:25;IV:20	CM + Getong Tongluo capsules (0.5g, bid)	CM	8w	①②④
Duan (2000)	38	40	54.2±8.2	52.6±9.45	19/19	22/18	NR	II:11;III:21;IV:6	II:10;III:25;IV:5	CM + puerarin injection (400mg)	CM	10d	①
Duan (2021)	34	34	65.89±4.34	65.99±4.31	19/15	20/14	2016 ESC guidelines	NR	NR	CM + Baoyuan decoction (200mL, bid)	CM	4w	①②③ ⑤⑦⑧
Fan et al. (2020)	63	63	66.0±3.7	67.0±3.6	33/30	36/27	China’s guideline (2018)	II:12;III:51	II:10;III:53	CM + Yangxinshi tablets (1.8g, tid)	CM	12m	①⑥⑧
Gao et al. (2021)	39	39	62.54±7.47	63.00±6.82	21/18	20/19	China’s guideline (2018)	II:4;III:27;IV:8	II:5;III:28;IV:6	CM + Yangxinshi tablets (1.8g, tid)	CM	6m	①④⑤⑥
Gong et al.(2006)	43	43	54±8	53±9	24/19	25/18	NR	III:57;IV:29		CM + puerarin injection (400mg)	CM	2w	①④
Huang et al. (2021)	47	47	68.21±5.43	66.94±5.17	29/18	31/16	China’s guideline (2018)	II:10;III:26;IV:11	II:13;III:25;IV:9	CM + Dange Wuling powder (150mL, bid)	CM	3m	①② ③④⑤⑥⑦⑧
Li (2017)	47	47	61.35±8.67	61.58±7.64	25/22	24/23	NR	II:20;III:18;IV:9	II:21;III:16;IV:10	CM + Yangxinshi tablets (1.8g, tid)	CM	4w	①②③④
Li et al. (2019)	60	60	66.6±12.5	64.9±12.3	34/26	26/34	China’s guideline (2014)	II:12;III:22;IV:26	II:9;III:24;IV:27	CM + Yangxinshi tablets (1.2g, tid)	CM	6m	①⑥⑦
Li et al. (2020)	100	100	69.37±5.42	69.85±5.74	54/46	57/43	China’s guideline (2014)	NR	NR	CM + Tongyang Lishui decoction (200mL, bid)	CM	2w	①④⑥
Li et al. (2021)	46	46	65.32±7.69	64.41±7.18	24/22	27/19	China’s guideline (2018)	II:17;III:29	II:19;III:27	CM + Yiqi Yangyin Tongmai decoction (200mL, bid)	CM	3m	①④⑥⑦⑧
Ma et al. (2017)	53	46	70.12±12.08	69.27±12.34	35/18	30/16	NR	II:15;III:21;IV:17	II:13;III:18;IV:15	CM + Xinshuning capsules (3.2g, bid)	CM	8w	①②③④⑤⑧
Ren et al. (2022)	76	76	71.73±0.50	71.25±1.00	39/37	40/36	China’s guideline (2018)	III:37;IV:39	III:40;IV:36	CM + Linggui Zhugan decoction (200mL, bid)	CM	4w	④⑦
Shi et al.(2008)	43	43	53.2±8.23		51/35		China’s guideline (2002)	III:57;IV:29		CM + puerarin injection (600mg)	CM	2w	①④
Tang et al.(2021)	60	60	75.2±11.9	76.7±13.6	36/24	40/20	China’s guideline (2018)	III:44;IV:46	III:40;IV:20	CM + Yangxinshi tablets (1.8g, tid)	CM	6m	①②④⑧
Wang (2022)	35	35	56.99±5.33	57.69±5.32	20/15	19/16	China’s guideline (2018)	II:17;III:18	II:19;III:16	CM + Getong Tongluo capsules (0.5g, bid)	CM	6m	①②③④
Zhang (2022)	30	30	68.46±8.08	68.67±6.63	18/12	22/8	China’s guideline (2018)	II:8;III:16;IV:6	II:9;III:16;IV:5	CM + Yixintong capsules (2.1g, tid)	CM	3m	①④
Zhao (2016)	49	49	59.2±11.7	58.7±12.6	30/19	28/21	China’s guideline (2007)	NR	NR	CM + puerarin injection (500mg)	CM	20d	①④

Abbreviations: C, control group; T, treatment group; M, male; F, female; NR, not reported; d, days; w, weeks; m, months; qd, quaque in die; bid, bis in die; tid, ter in die; CM, conventional medicine; NYHA, New York Heart Association; Outcomes:

①left ventricular ejection fraction

②left ventricular end-diastolic diameter

③left ventricular end-systolic diameter

④the clinical efficacy rate

⑤N-terminal pro-brain natriuretic peptide

⑥6-min walk test

⑦the TCM syndrome score efficacy

⑧adverse events.

### Risk of bias

**Fig 2:**
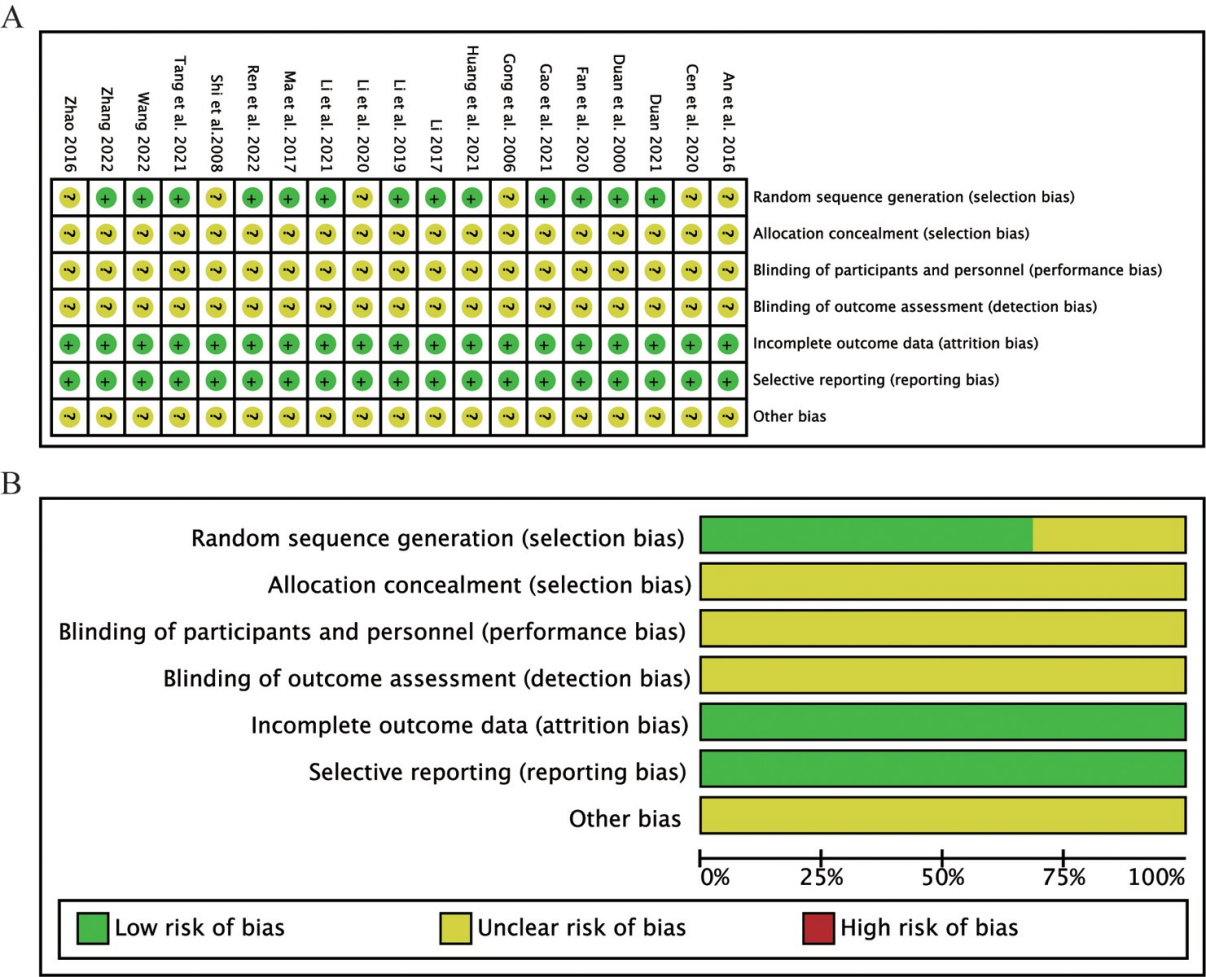
Risk of bias diagram and summary. (A) Risk of bias summary. (B) Risk of bias graph.

**Fig 2** showed the quality assessment result. Only 13 studies [13, 18–20, 22, 24–31] reported the randomization method and their random sequences were generated by random number tables. However, 6 studies [14–17, 21, 23] did not report the randomization method, so their risk levels were unclear. Blinding and allocation concealment procedures were not described in any of the 19 studies. The risk of other biases was unclear.

### Primary outcomes

#### LVEF

**Fig 3:**
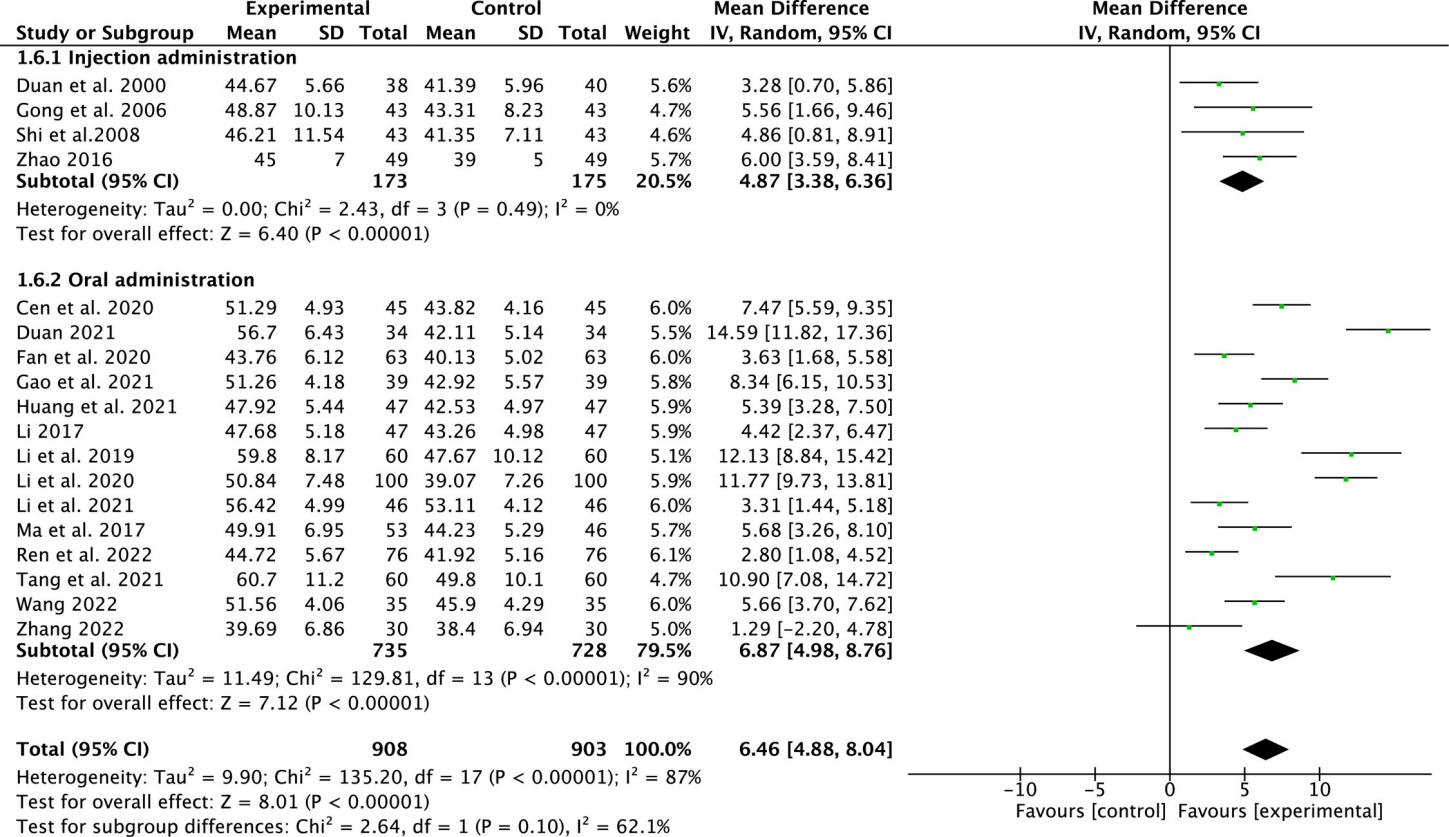
Forest plot of LVEF.

18 studies [13–16, 18–31] that included 1,811 patients reported LVEF. Due to the high heterogeneity (*P*<0.00001, *I*^2^ = 87%), a random-effect model was applied. Compared to conventional medicine, pueraria combination therapy markedly improved LVEF (MD = 6.46, 95% CI, 4.88 to 8.04, *P* < 0.00001, Fig 3). Subgroup analysis indicated that pueraria oral administration and injection could better elevate LVEF than conventional medicine (MD = 4.87, 95% CI, 3.38 to 6.36; MD = 6.87, 95% CI, 4.98 to 8.76, for pueraria injection and oral administration, respectively, Fig 3).

#### LVEDD

**Fig 4:**
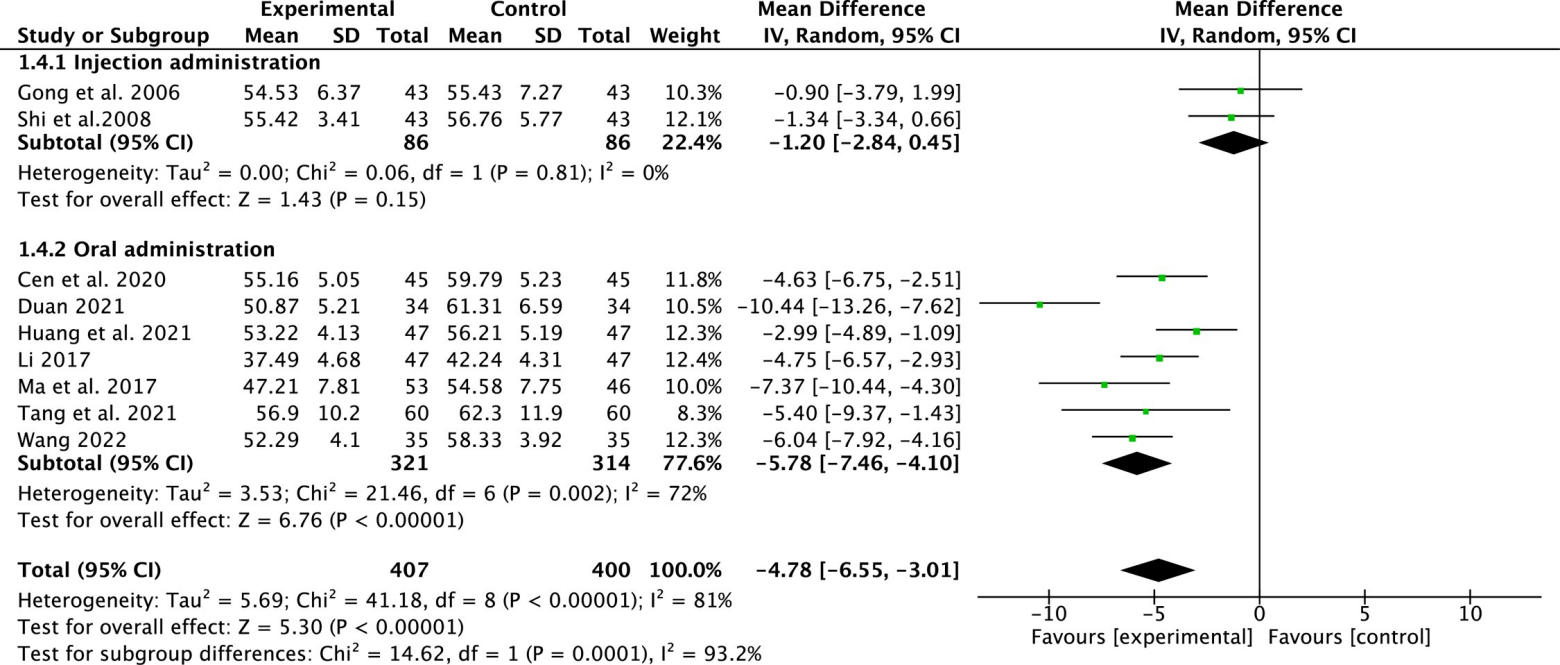
Forest plot of LVEDD.

9 studies [14, 15, 18, 19, 21, 24, 26, 28, 30] that included 807 patients reported LVEDD. Due to the high heterogeneity (*P*<0.00001, *I*^2^ = 81%), a random-effect model was applied. Compared to conventional medicine, pueraria combination therapy significantly decreased LVEDD (MD = -4.78, 95% CI, -6.55 to -3.01, *P* < 0.00001, Fig 4). Subgroup analysis indicated that pueraria oral administration could better improve LVEDD (MD = -5.78, 95% CI, -7.46 to -4.10, *P* < 0.00001, Fig 4).

#### LVESD

**Fig 5:**
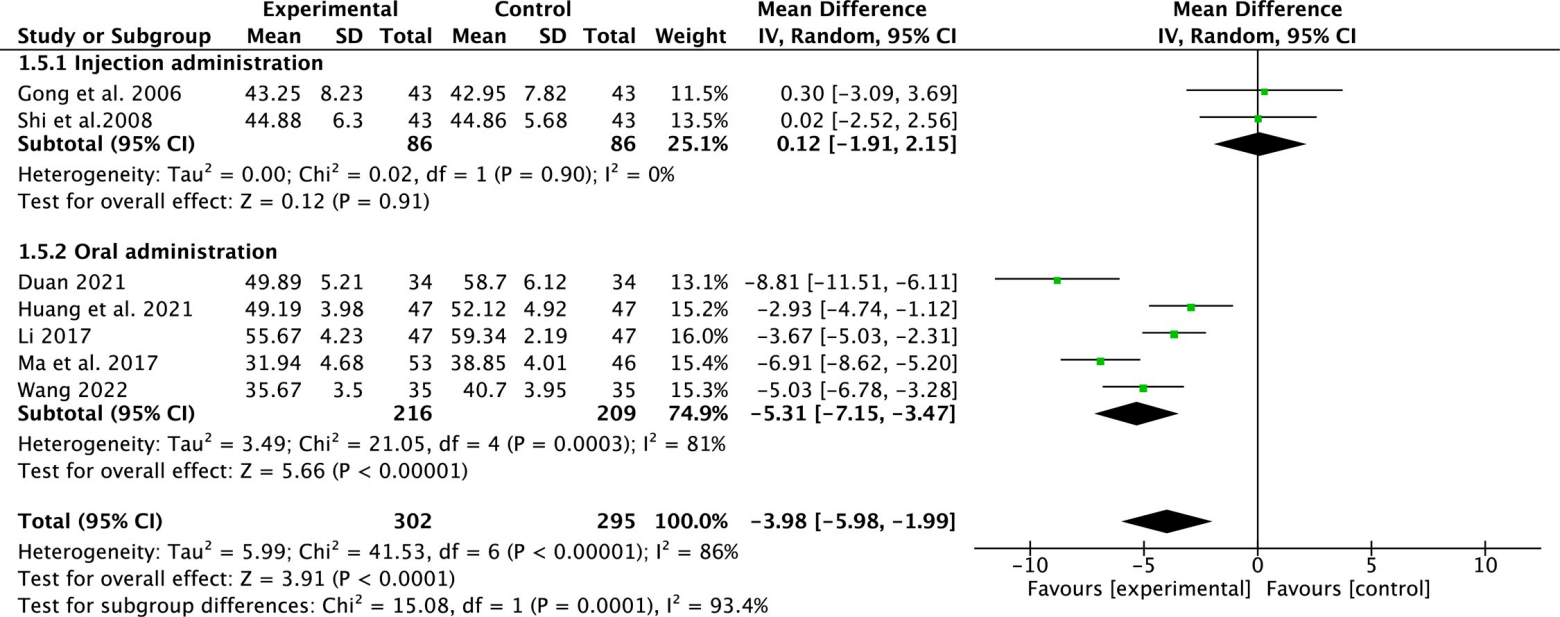
Forest plot of LVESD.

7 studies [14, 15, 18, 19, 24, 26, 30] that included 597 patients reported LVESD. Due to the high heterogeneity (*P*<0.00001, *I*^2^ = 86%), a random-effect model was applied. Compared to conventional medicine, pueraria combination therapy significantly decreased LVESD (MD = -3.98, 95% CI, -5.98 to -1.99, *P* < 0.00001, Fig 5). Subgroup analysis indicated that pueraria oral consumption could better improve LVESD (MD = -5.31, 95% CI, -7.15 to -3.47, *P* < 0.00001, Fig 5).

### Secondary outcomes

#### Clinical efficacy rate

**Fig 6:**
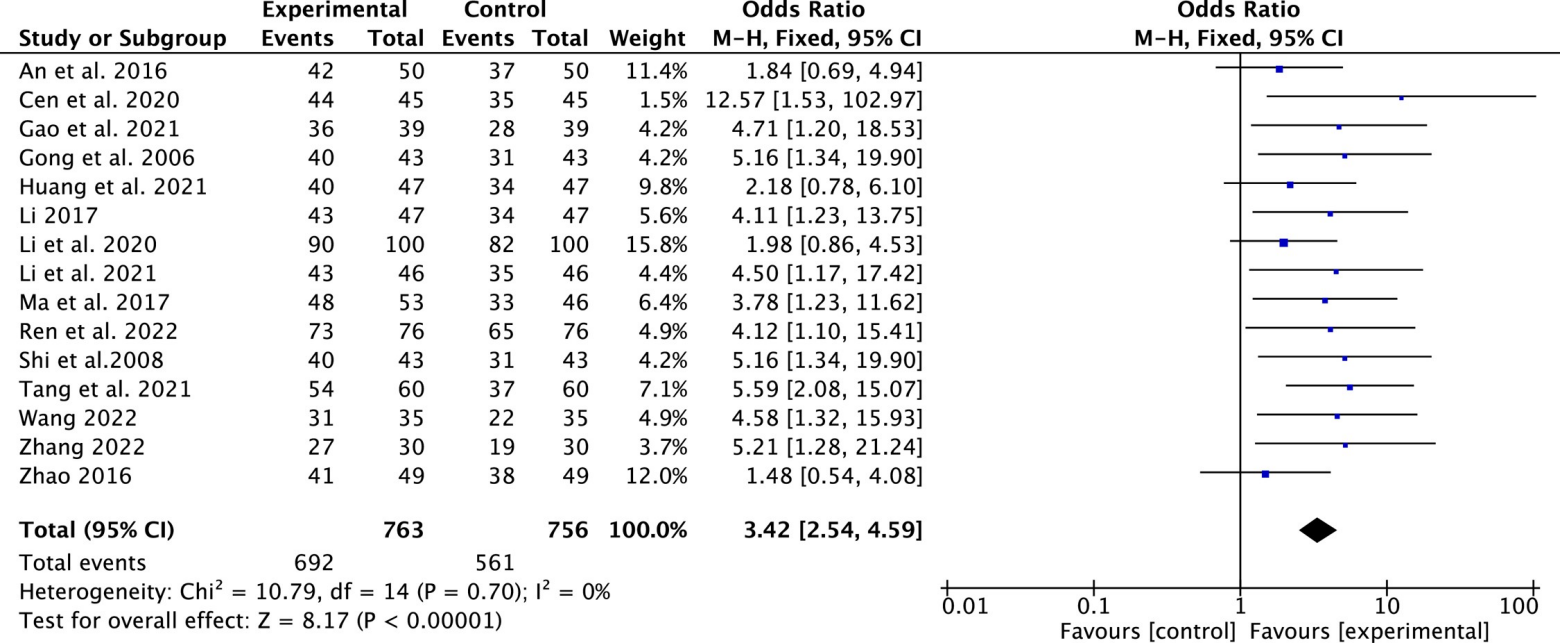
Forest plot of clinical efficacy rate.

15 studies [14–19, 21, 23, 25–31] that included 1,519 patients reported clinical efficacy rate. Due to the low heterogeneity (*P* = 0.70, *I*^2^ = 0%), a fixed-effect model was applied. Compared to conventional medicine, pueraria combination therapy significantly improved the clinical efficacy rate (RR = 3.42, 95% CI, 2.54 to 4.59, *P* < 0.00001, Fig 6).

#### NT-proBNP

**Fig 7:**
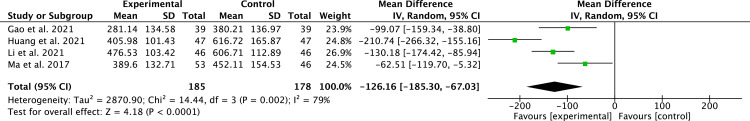
Forest plot of NT-pro BNP.

4 studies [19, 25–27] that included 363 patients reported NT-proBNP. Due to the high heterogeneity (*P* = 0.002, *I*^2^ = 79%), a random-effect model was applied. Compared to conventional medicine, pueraria combination therapy significantly reduced NT-proBNP (MD = -126.16, 95% CI, -185.30 to -67.03, *P* < 0.0001, Fig 7).

#### 6-MWT

**Fig 8:**
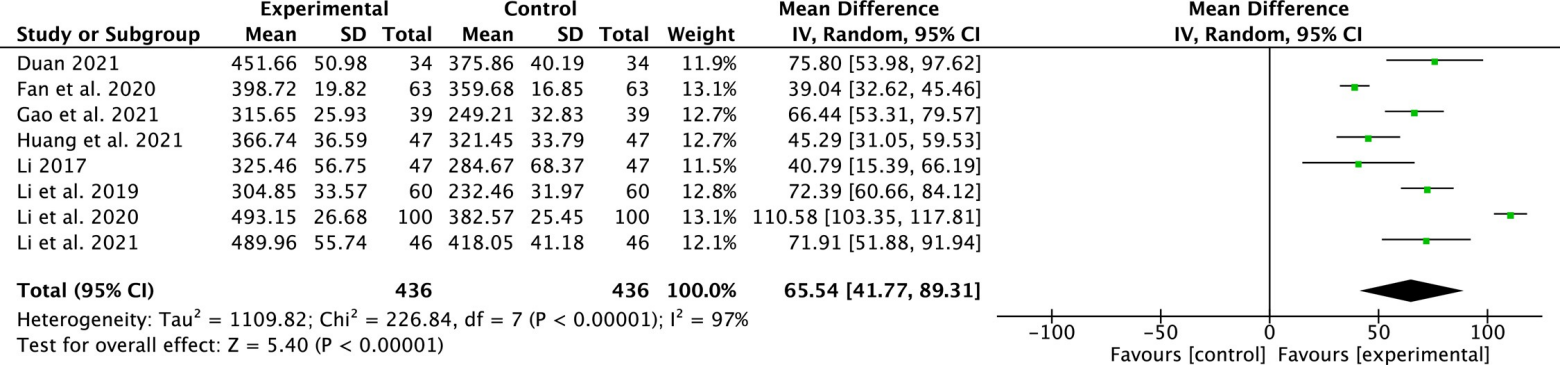
Forest plot of 6-MWT.

8 studies [18, 20, 22–27] that included 872 patients reported 6-MWT. Due to the high heterogeneity (*P* < 0.00001, *I*^2^ = 97%), a random-effect model was applied. Compared to conventional medicine, pueraria combination therapy significantly improved 6-MWT (MD = 65.54, 95% CI, 41.77 to 89.31, *P* < 0.00001, Fig 8).

#### TCM syndrome score efficacy

**Fig 9:**
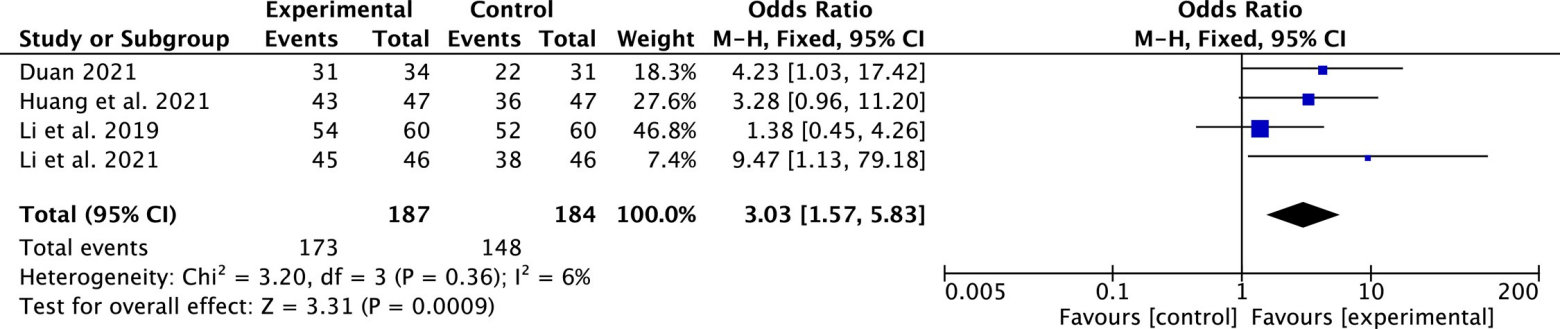
Forest plot of TCM syndrome score efficacy.

4 studies [20, 24, 26, 27] that included 371 patients reported the TCM syndrome score efficacy. Due to low heterogeneity (*P* = 0.36, *I*^2^ = 6%), we applied a fixed-effect model. Compared to conventional medicine, pueraria combination therapy significantly improved the TCM syndrome score efficacy (RR = 3.03, 95% CI, 1.57 to 5.83, *P* = 0.0009, Fig 9).

#### Adverse drug events

Adverse drug events were reported in 10 studies [13–16, 19, 22, 24, 26–28], 4 [13–15, 28] of which reported no adverse events occurred. 7 studies [16, 19, 22, 24, 26, 27] that included 577 patients reported a total of 6.51% (19/292) adverse events in pueraria combination therapy group and 12.63% (36/285) adverse events in the conventional medicine group. Pueraria combination therapy could cause dizziness, headache, skin rash, astriction, gastrointestinal upset, blood potassium increased, respiratory adverse reactions, and re-hospitalization for patients with HFrEF. Similarly, using conventional medicine alone could cause dizziness, headache, hypotension, skin rash, astriction, arrhythmias, gastrointestinal upset, respiratory adverse reactions, and re-hospitalization. No serious adverse events were reported in both groups. Compared to conventional medicine, pueraria combination therapy was detected without a clear predominance on adverse events (RR = 0.59, 95% CI, 0.22 to 1.54, *P* = 0.28). Additionally, 1 study [26] reported no statistical difference in re-hospitalization and mortality between the two groups during a 6-month follow-up period. **Table 2** provided detailed information.

**Table 2:** The incidence rate of adverse events.

Type	Study	The number of adverse events	
		Treatment group	Control group
Dizziness	(Duan, 2021; Li et al., 2021)	2	1
Headache	(Ma et al., 2017)	1	1
Hypotension	(Ma et al., 2017)	0	2
Skin rash	(Ma et al., 2017)	1	1
Astriction	(Duan, 2021)	1	1
Arrhythmias	(Zhao, 2016)	0	6
Gastrointestinal upset	(Duan, 2021; Huang et al., 2021; Li et al., 2021; Ma et al., 2017; Zhao, 2016)	7	13
Blood potassium increased	(Li et al., 2021)	1	0
Respiratory adverse reactions	(Zhao, 2016)	3	3
Re-hospitalization	(Fan et al., 2020)	3	8
Total events	—	19/292	36/285
Incidence rate	—	6.51%	12.63%

#### Evaluation of publication bias

**Fig 10:**
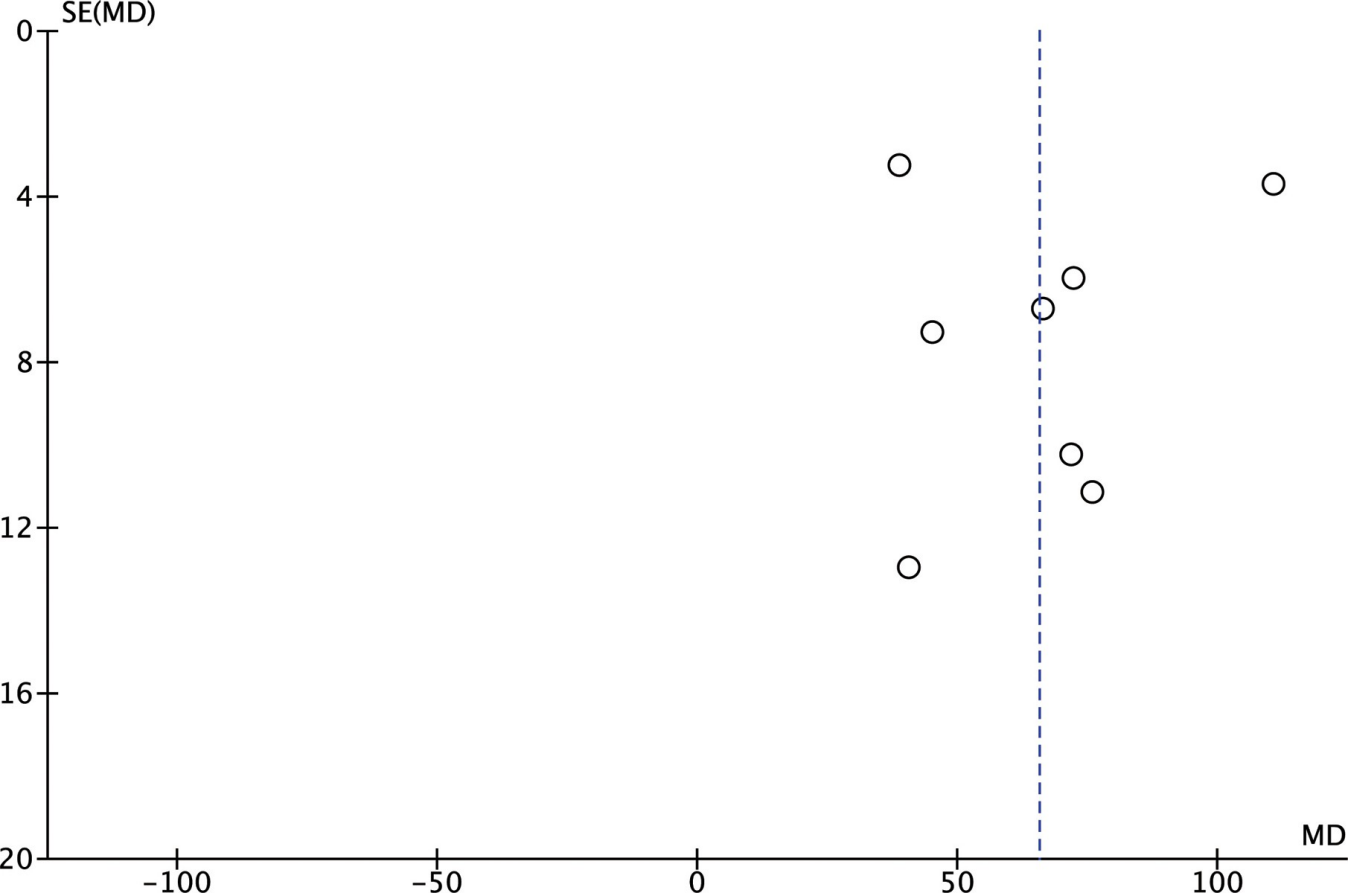
Funnel plot of 6-MWT.

Evaluation of publication bias was conducted by comparing 6-MWT. The funnel plot displayed that a slight asymmetry existed, indicating that there appeared to be a slight publication bias among the included studies (Fig 10). We analyzed the sources of publication bias by excluding studies one by one. Nevertheless, this method could not completely eliminate publication bias. Thus, we supposed that publication bias rooted in the fact that all included studies were conducted in China.

## Discussion

### Summary of findings

HFrEF remains a major global public health issue and is a leading cause of medical hospital admissions for individuals over the age of 60 [38–40]. While various medications aimed at improving ventricular remodeling and heart failure have been developed, they have not yielded entirely satisfactory results [41–43]. Currently, several drugs have been used clinically to treat LV remodeling, including diuretics, beta-blockers, spironolactone, angiotensin receptor blockers (ARBs), and Dagglizin. However, these drugs only marginally improve symptoms, slightly mitigate LV remodeling, modestly prolong the life of HFrEF patients [44]. The available drugs for LV remodeling are inadequate to meet current medical needs, and are restricted in their widespread clinical use due to adverse reactions, including dry cough, angioedema, hyperkalemia, low blood pressure, and irreversible kidney failure [45]. Due to the adverse events and limited therapeutic effects of western medicine, it is urgent to explore alternative and complementary therapies for ameliorating LV remodeling in HFrEF patients. In our study, it was found that compared with conventional medicine alone, combination therapy of Chinese herbal pueraria and conventional medicine had more benefit for the clinical outcomes of LV remodeling in HFrEF.

Pueraria is one of the most extensively utilized Chinese herbs. It is commonly employed as an anti-heart failure, anti-ventricular remodeling, liver protector, and kidney protector [10–12]. Puerarin from pueraria is demonstrated to alleviate heart failure and ventricular remodeling [10]. Puerarin can reverse ventricular remodeling induced by multiple stimuli [12]. Pueraria extract can ameliorate alcoholic liver injury by recovering liver pathological changes, inhibiting Kupffer cells activation, and downregulating hepatic TNF-α expression [46]. Puerarin exhibits hepatoprotection through the regulation of mTOR signaling pathway [8]. Puerarin also exerts renoprotection via regulating NF-κB p65/STAT3 and TGFβ1/Smads pathways [9].

The most commonly used monitoring variables for LV remodeling are LVEF, LVEDD, and LVESD [5]. In our study, pueraria combination therapy provided superior advantages in ameliorating LV remodeling parameters (LVEF, LVEDD, and LVESD). Subgroup analysis indicated that pueraria combination therapy improved LVEF, regardless of whether puerarin is taken orally or injected. The benefit of pueraria combination therapy in LV remodeling in HFrEF patients was evident regardless of subgroup analysis. This is attributed to pueraria’s active components that can reverse ventricular remodeling [12]. In addition, we also observed that pueraria combination therapy had betterments in cardiac function (6-MWT and NT-proBNP), clinical efficacy rate, and TCM syndrome score efficacy.

In terms of clinical safety, 6.51% (19/292) of adverse events occurred in pueraria combination therapy group, while 12.63% (36/285) in conventional medicine group. This is attributed to pueraria and its active components that have hepatoprotection and renoprotection [8–9]. Due to the fact that only six out of nineteen included studies had reported adverse events (dizziness, headache, hypotension, skin rash, astriction, arrhythmias, gastrointestinal upset, blood potassium increased, respiratory adverse reactions, and re-hospitalization due to cardiac insufficiency) [16, 19, 22, 24, 26, 27], more studies would be required to validate the safety of pueraria combination therapy in the treatment of HFrEF in future.

### Strengths and limitations

This study is the first meta-analysis to evaluate the effect of pueraria on left ventricular remodelling in HFrEF patients. It contributes to guiding clinical medication to ameliorate cardiac function and reverse left ventricular remodeling in HFrEF. By adhering to the PRISMA checklist and Cochrane Handbook, our study draws quantitative conclusions based on scientific and rigorous research. Ultimately, our combined results suggest that pueraria combination therapy can more effectively improve left ventricular remodeling and enhance clinical efficacy in patients with HFrEF.

However, several limitations existed in our review. Firstly, not all of the included RCTs described the allocation concealment and blinding methods. This may have resulted in potential selective bias. Secondly, despite our comprehensive search, all the included RCTs in our study were carried out in China. Thus, the findings may not be generalizable. Thirdly, none of the included RCTs used placebos, which is a potential limitation. Therefore, our findings need to be supported by more well-designed RCTs. Fourthly, most of the included studies did not describe the follow-up periods, which may have inadequately evaluated the results. Fifthly, due to the included RCTs were conducted between 2000 and 2022, the diagnostic criteria of different periods were inconsistent, which is a possible limitation. Despite these limitations, our manuscript is the first meta-analysis to assess pueraria combination therapy for left ventricular remodeling in HFrEF patients. It will draw great attention from researchers and doctors to the potential benefits of pueraria combination therapy for HFrEF.

## Conclusion

According to current evidence, pueraria combined with conventional medicine has superiority over conventional medicine alone in reversing LV remodeling (LVEF, LVEDD, and LVESD), ameliorating cardiac function (6-MWT and NT-proBNP), improving clinical efficacy rate and TCM syndrome score efficacy, as well as good safety in treating HFrEF. However, given some limitations, our conclusion needs to be verified by more high-quality, more prudent clinical studies in the future.

## Supporting information

S1 Text Search strategies. (DOCX)

S2 Text Risk of bias of included studies. (DOCX)

S1 Checklist PRISMA 2020 checklist. (DOCX)
