# Supplementary material for: Effect of pueraria on left ventricular remodelling in HFrEF: A systematic review and meta-analysis
Source: PLoS One. 2023 Dec 4;18(12):e0295344. doi: 10.1371/journal.pone.0295344 (PMC10695375; doi:10.1371/journal.pone.0295344)
Supplement: S1 Text — (DOCX) [file pone.0295344.s001.docx]

# Supplementary Text S1. Search strategies.

| Electronic databases | Search strategies |
| --- | --- |
| Pubmed | #1 heart failure [MeSH Terms] OR heart failure [Title/Abstract] OR heart dysfunction [Title/Abstract] OR ventricular dysfunction [MeSH Terms] OR ventricular dysfunction [Title/Abstract] OR heart insufficiency [MeSH Terms] OR heart insufficiency [Title/Abstract] |
|  | #2 puerarin [All Fields] OR pueraria [All Fields] OR kudzu [All Fields] OR pueraria lobata [All Fields] |
|  | #3 randomized controlled trial [Publication Type] OR clinical trial [Publication Type] OR randomized controlled trial [Mesh Terms] OR randomized controlled trial [Title/Abstract] OR double-blind [Title/Abstract] OR single-blind [Title/Abstract] OR random [Title/Abstract] OR trial [Title/Abstract] |
|  | #4 #1 AND #2 AND #3 |
| EMBASE | #1 heart failure: ti,ab OR heart dysfunction: ti,ab OR ventricular dysfunction: ti,ab OR heart insufficiency: ti,ab |
|  | #2 pueraria: ti,ab OR puerarin: ti,ab OR kudzu: ti,ab OR pueraria lobata: ti,ab |
|  | #3 random: ti,ab OR trial: ti,ab |
|  | #4 #1 AND #2 AND #3 |
| Cochrane Library | #1 (heart failure): ti,ab,kw OR (heart dysfunction): ti,ab,kw OR (ventricular dysfunction): ti,ab,kw OR (heart insufficiency): ti,ab,kw |
|  | #2 (pueraria): ti,ab,kw OR (puerarin): ti,ab,kw OR (kudzu): ti,ab,kw OR (pueraria lobata): ti,ab,kw |
|  | #3 #1 AND #2 |
| ClinicalTrials.gov | #1 (heart failure OR heart dysfunction OR ventricular dysfunction OR heart insufficiency): Condition or disease |
|  | #2 (pueraria OR puerarin OR kudzu OR pueraria lobata): Intervention/Treatment |
|  | #3 #1 AND #2 |
