# Supplementary material for: Effect of pueraria on left ventricular remodelling in HFrEF: A systematic review and meta-analysis
Source: PLoS One. 2023 Dec 4;18(12):e0295344. doi: 10.1371/journal.pone.0295344 (PMC10695375; doi:10.1371/journal.pone.0295344)
Supplement: S2 Text — (DOCX) [file pone.0295344.s002.docx]

# Supplementary Text S3. Risk of bias of included studies.

| **Study ID** | **Random sequence generation** | **Allocation concealment** | **Blinding of participants and personnel** | **Blinding of outcome assessment** | **Incomplete outcome data** | **Selective reporting** | **Other bias** |
| --- | --- | --- | --- | --- | --- | --- | --- |
| An et al. (2016) | Unclear risk | Unclear risk | Unclear risk | Unclear risk | Low risk | Low risk | Unclear risk |
| Cen et al. (2020) | Unclear risk | Unclear risk | Unclear risk | Unclear risk | Low risk | Low risk | Unclear risk |
| Duan et al. (2000) | Unclear risk | Unclear risk | Unclear risk | Unclear risk | Low risk | Low risk | Unclear risk |
| Duan (2021) | Low risk | Unclear risk | Unclear risk | Unclear risk | Low risk | Low risk | Unclear risk |
| Fan et al. (2020) | Low risk | Unclear risk | Unclear risk | Unclear risk | Low risk | Low risk | Unclear risk |
| Gao et al. (2021) | Low risk | Unclear risk | Unclear risk | Unclear risk | Low risk | Low risk | Unclear risk |
| Gong et al. (2006) | Unclear risk | Unclear risk | Unclear risk | Unclear risk | Low risk | Low risk | Unclear risk |
| Huang et al. (2021) | Low risk | Unclear risk | Unclear risk | Unclear risk | Low risk | Low risk | Unclear risk |
| Li (2017) | Low risk | Unclear risk | Unclear risk | Unclear risk | Low risk | Low risk | Unclear risk |
| Li et al. (2019) | Low risk | Unclear risk | Unclear risk | Unclear risk | Low risk | Low risk | Unclear risk |
| Li et al. (2020) | Unclear risk | Unclear risk | Unclear risk | Unclear risk | Low risk | Low risk | Unclear risk |
| Li et al. (2021) | Low risk | Unclear risk | Unclear risk | Unclear risk | Low risk | Low risk | Unclear risk |
| Ma et al. (2017) | Low risk | Unclear risk | Unclear risk | Unclear risk | Low risk | Low risk | Unclear risk |
| Ren et al. (2022) | Low risk | Unclear risk | Unclear risk | Unclear risk | Low risk | Low risk | Unclear risk |
| Tang et al. (2021) | Low risk | Unclear risk | Unclear risk | Unclear risk | Low risk | Low risk | Unclear risk |
| Shi et al. (2008) | Unclear risk | Unclear risk | Unclear risk | Unclear risk | Low risk | Low risk | Unclear risk |
| Wang (2022) | Low risk | Unclear risk | Unclear risk | Unclear risk | Low risk | Low risk | Unclear risk |
| Zhang (2022) | Low risk | Unclear risk | Unclear risk | Unclear risk | Low risk | Low risk | Unclear risk |
| Zhao (2016) | Unclear risk | Unclear risk | Unclear risk | Unclear risk | Low risk | Low risk | Unclear risk |
